# First Confirmed Record of a Bull Shark in Lake Gatun, the Freshwater Body of the Panama Canal

**DOI:** 10.1002/ece3.73114

**Published:** 2026-02-23

**Authors:** Gustavo A. Castellanos‐Galindo, D. Ross Robertson, Victor Bravo, Kristin Saltonstall, Phillip Sanchez, Lucia Morales, Richard Cahill, Mark E. Torchin

**Affiliations:** ^1^ Leibniz Institute of Freshwater Ecology and Inland Fisheries (IGB) Berlin Germany; ^2^ Freie Universität Berlin Berlin Germany; ^3^ Smithsonian Tropical Research Institute Panama City Panama; ^4^ Leibniz Centre for Tropical Marine Research (ZMT) Bremen Germany; ^5^ Panama Tarpon Conservation Panama City Panama

**Keywords:** bull shark, eastern Pacific, fish invasions, interoceanic movement, Lake Gatun, Panama

## Abstract

Bull Sharks are circumtropical top predators able to tolerate a wide range of salinity conditions that include freshwater. In several areas of Central America this species is known to migrate upstream in rivers and is commonly found in freshwater. The Panama Canal, an engineered system critical for global shipping, has experienced repeated marine fish incursions into Lake Gatun, the freshwater portion of the system, since it opened over 100 years ago. With increased numbers of species and abundance of these marine migrants into the system it is surprising that no credible reports of Bull Sharks have been made to date. Here we present the first confirmed report of a Bull Shark captured in Lake Gatun, 30 km from the Pacific entrance of the Canal. Analyzing its DNA barcode and vertebral morphometrics and chemistry, we were able to infer the origin (Pacific Ocean), the total length and age (120–150 cm, 2–3 year old) and likely pupping of this shark in low salinity areas adjacent to the Canal. The recent capture of more bull sharks by the artisanal fisher who collected the study shark and a video of sharks at the seaward entrance to the new Pacific locks indicates that there is the potential for increased contact between Pacific and Atlantic Bull Shark populations through the Panama Canal.

## Graphical abstract

The Panama Canal, an engineered system critical for global shipping, has experienced repeated marine fish incursions into its freshwater portion since it opened > 100 years ago. With increased numbers of species and abundance of these marine migrants into the system, surprisingly, no credible reports of Bull Sharks have been made to date. Here we present the first confirmed report of a Bull Shark inside the Panama Canal, 30 km from its Pacific entrance, and analyze the potential implications of this finding.

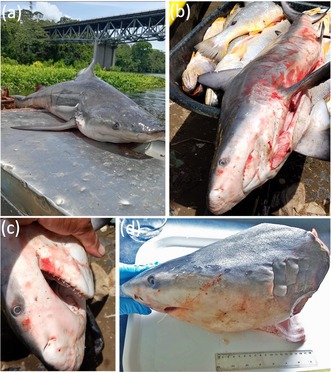

## 1 Introduction

Shifts in fish communities within the Panama Canal have occurred since its operation began in 1914 (Hildebrand 1939). However, over the last decade, numerous species of marine bony fishes have been observed in the freshwater of the Panama Canal, and in some locations, catches of these species outnumber the freshwater species (Sharpe et al. 2017; Castellanos‐Galindo et al. 2020, 2025). Notably, there has been a shift in the trophic structure of the food web, with upper trophic levels now dominated by large marine predators (Castellanos‐Galindo et al. 2025). Here we report the first confirmed record of a Bull Shark ( *Carcharhinus leucas* ), a top‐order marine carnivore, in the freshwater portion of the Panama Canal.

The Bull Shark (in Spanish: tiburón toro), *Carcharhinus leucas* (Valenciennes, 1839), is a large (up to 4 m total length, > 300 kg weight), circumtropical predator that mainly inhabits coastal areas (Rigby et al. 2021). This species is well known for its capacity to tolerate a wide range of salinity conditions that include freshwater (Thomerson et al. 1977). It is found in rivers and freshwater lakes throughout its geographic range and can penetrate rivers as much as ~4000 km from the coast (e.g., the Amazon River; Thorson 1972; Gausmann 2021). Recently, Devloo‐Delva et al. (2023), using SNP markers and full mitogenome data, identified four genetic clusters in Bull Shark populations around the world (Eastern Pacific, Western Atlantic, Eastern Atlantic and Indo‐West Pacific). In particular, the Eastern Pacific cluster was strongly differentiated from the Western Atlantic cluster (Devloo‐Delva et al. 2023), indicating that the Bull Shark populations from those regions have been demographically isolated for a long time. There are various records of Bull Sharks found in freshwater ecosystems in Central America (Gausmann et al. 2025). Probably the most well‐known location is Lake Nicaragua. Recorded as early as the 16th Century, the Bull Shark population in this system was first thought to be landlocked and considered a different species ( *Eulamia nicaraguensis* ) by Gill and Bransford (1877) and other authors. Later, Bigelow and Schroeder (1961) found no morphological characteristics that differentiate Bull Sharks from Lake Nicaragua and marine *C. leucas* . After establishing that Bull Sharks can navigate the ~200 km of the San Juan River from the Caribbean, Thorson et al. (1966) showed that the population in Lake Nicaragua is not landlocked. In Panama in 1981, Vásquez Montoya and Thorson (1982), found three dead mature *C. leucas* females in Lake Bayano, an artificial reservoir located 70 km east of the Panama Canal. That lake was created in 1976 by damming of the Bayano River, which drains to the Pacific coast. The authors hypothesized that those individuals were landlocked by the creation of the dam at Bayano.

The Panama Canal has been a major artery for global maritime traffic since 1914. The construction of the Canal involved the damming of the Chagres River, which previously discharged into the Caribbean, and inundated > 400 km^2^ of tropical rain forest to create a large freshwater reservoir, Lake Gatun. In addition, a system of locks on the Pacific Ocean and the Caribbean Sea allows the passing of vessels through the lake. Construction on the Pacific side also involved substantial changes to the coastal estuaries in that area, with entrances to small rivers becoming part of the Pacific entrance to the Canal. Rivers and estuaries on both sides of the Canal have conditions (highly variable salinity, shallow and nutrient‐rich waters) conducive to usage by Bull Sharks, which are commonly found in coastal waters of both the Western Atlantic and Eastern Pacific oceans.

Here we present the first confirmed report of a Bull Shark inside the freshwater portion of the Panama Canal. Because this species is found on both Pacific and Atlantic coasts, our finding has implications for the possibility of interoceanic migrations of populations of this species and others and is consistent with reports of an abundance of various marine bony fishes in Lake Gatun.

## 2 Methods

**FIGURE 1:**
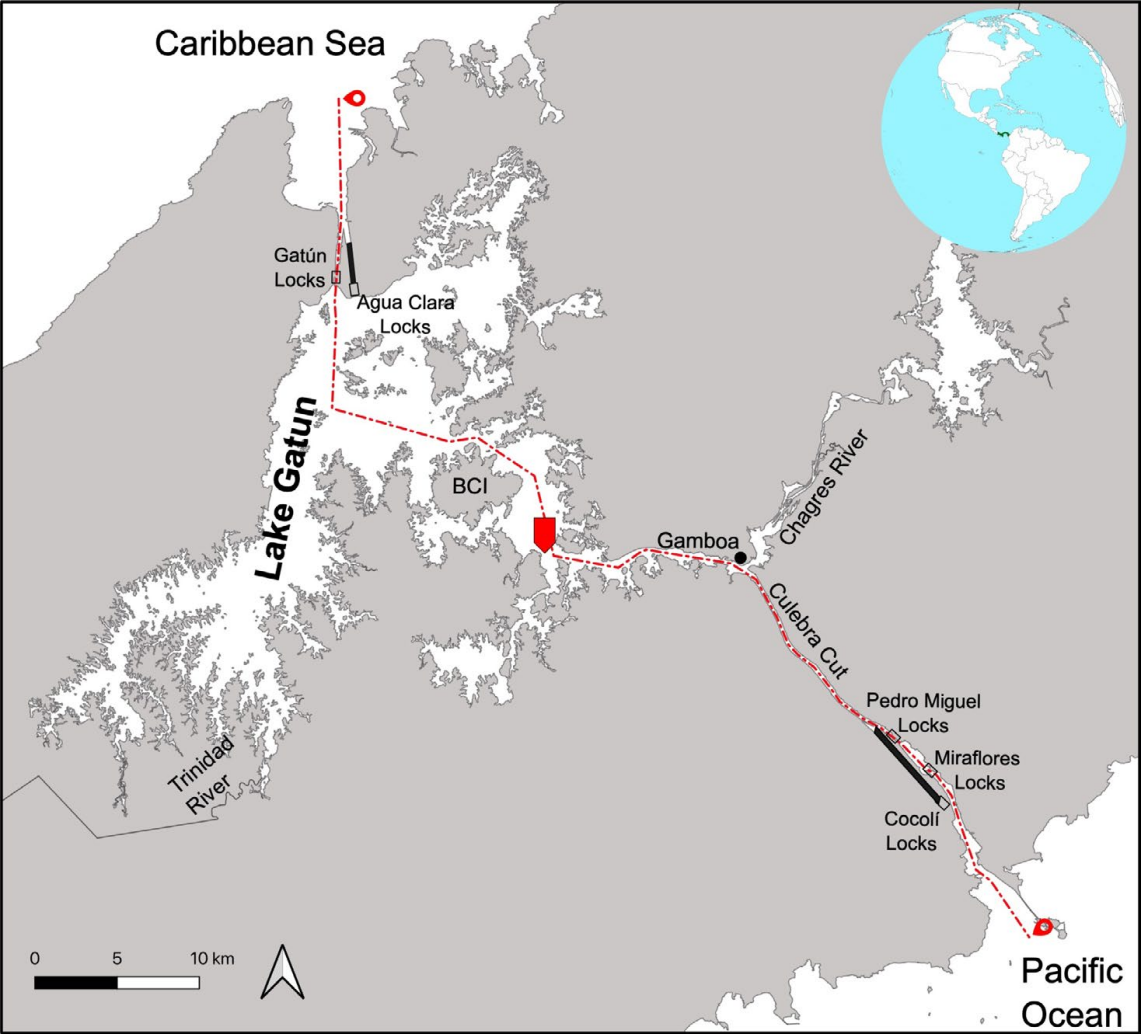
Location (red pointed polygon in center of map) of the Bull Shark captured inside the freshwater portion of the Panama Canal. The red dotted line indicates the navigation channel of the Canal and the red symbols at the ends show its terminus in each ocean. BCI refers to Barro Colorado Island.

On March 28th, 2025 we were informed by local artisanal fishers about the inadvertent capture of a shark inside Lake Gatun. We were only able to secure the head of the captured specimen because the rest of the body was sold before we reached the landing site. However, the fisher provided several photographs of the shark and information on the capture location. This fisher and others at the same base routinely fish near the boat navigation channel of Lake Gatun between Gamboa and Barro Colorado Island (BCI; Figure 1). They have traditionally targeted introduced Nile Tilapia ( *Oreochromis niloticus* ), and more recently, large marine fishes such as the Pacific White Corvina ( *Cynoscion albus* ) using gillnets.

**FIGURE 2:**
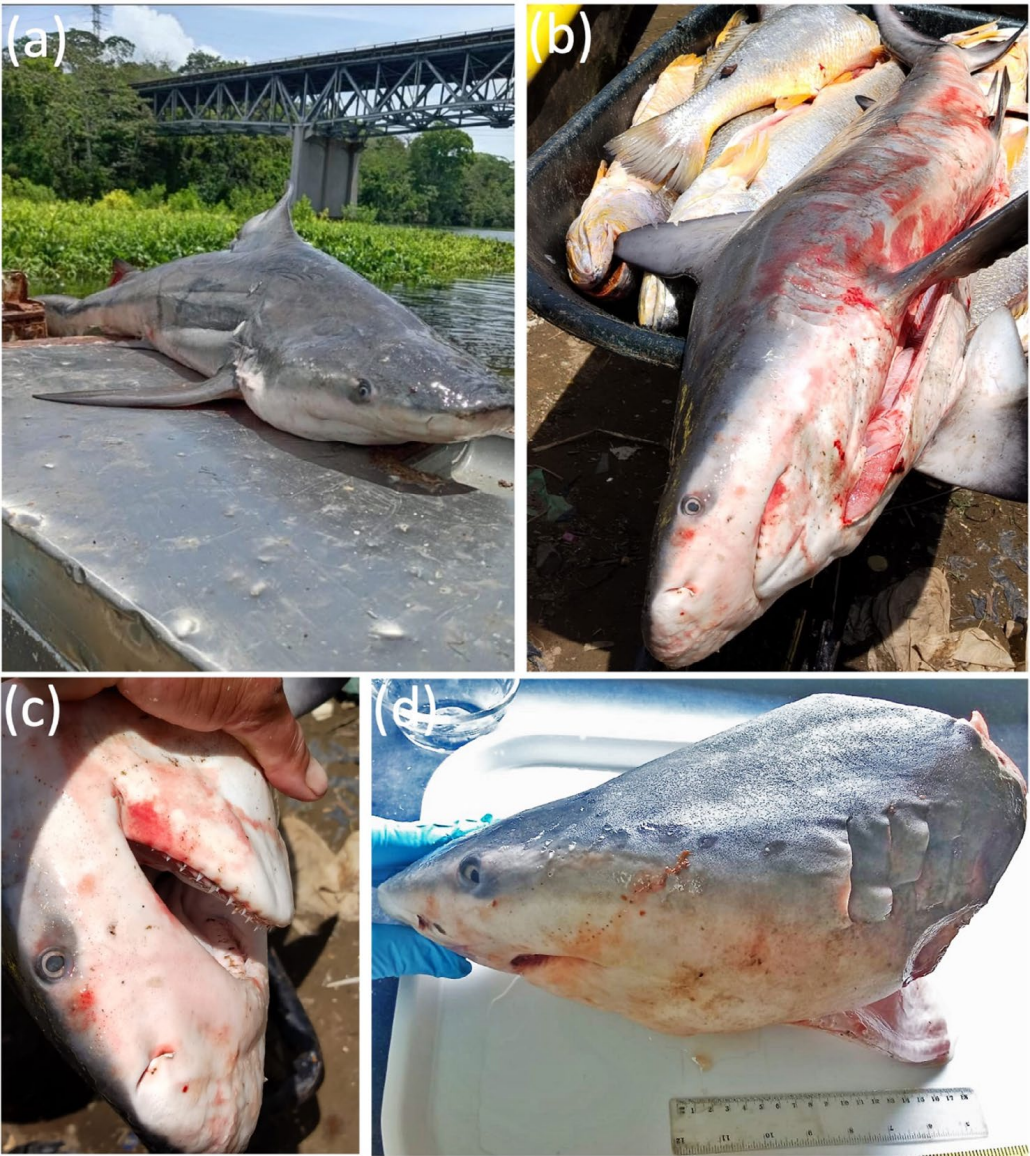
Images of a Bull Shark captured inside the freshwater portion of the Panama Canal in Central America. (a–c) Photographs of the Bull Shark taken at the landing location; and (d) Lateral view of the Bull Shark head taken in the laboratory. The shark was caught by an artisanal fisher when fishing for Pacific White Corvinas ( *Cynoscion albus* ), see yellow‐bellied grayish fish in (b) with gillnets inside Lake Gatun.

We used two methods to estimate the total length (TL) of the shark. The first method involved measuring the lateral view of the shark head on a photograph (snout tip to the end of the last gill slit according to Compagno 2001; Figure 2d) and using head to total length relationships in photographs of lateral views of specimens of entire Bull Sharks to estimate TL of our specimen. The second method involved vertebrae morphometrics and age determination to estimate the TL and age of the individual. The first ten vertebrae posterior to the cranium were removed, separated, and soaked in lightly boiling water to remove all connective tissue. The largest fully intact vertebra was sectioned along the frontal plane at a thickness of 0.75 mm. The sectioned vertebra was mounted onto a petrographic slide and viewed under transmitted light to expose band pairs.

To identify if this shark originated from the Pacific or Caribbean side of the Canal, DNA was extracted from muscle tissue using the Qiagen Blood and Tissue DNA extraction kit, according to the manufacturer's instructions. A 655 bp portion of the mitochondrial COI locus was PCR amplified using primer set BCH and BCL (Baldwin et al. 2009). The resulting amplicon was sequenced using BigDye chemistry on an ABI 3500XL machine (Applied Biosystems) at the STRI Naos Molecular Laboratory. Sequences were aligned and cleaned, verified using BLAST, and an Unweighted Pair Group Method with Arithmetic Mean (UPGMA) consensus tree (1000 bootstraps) comparing this sequence with other *C. leucas* sequences obtained from NCBI was constructed, as implemented in Geneious 2025.0.3. A sequence of the Pigeye Shark *Carcharhinus amboinensis* , which is the closest relative of *C. leucas* (see Brée et al. 2022), was used to root the UPGMA tree.

Vertebral chemistry was assessed along a lifetime transect from the vertebral centra to edge along the corpus calcareum, in the direction of radial growth using an ESI NWR193 excimer laser ablation system (193 nm, 4 ns pulse) coupled to an Agilent 8900 inductively coupled plasma mass spectrometer (LA‐ICPMS). Before analysis, the transect region was pre‐ablated to remove shallow surface contaminants by running a 20 × 125 μm spot at a 50 μm/s scan rate and 3 J/cm^2^ fluence up the corpus calcareum. Concentrations of five elemental masses that correlate with ambient salinity were quantified with 10 ms (^43‐44^Ca), 20 ms (^88^Sr), and 100 ms integration times (^137‐138^Ba). Measured intensities were converted to elemental concentrations (ppm) using iolite software (Paton et al. 2011), using ^43^Ca as the internal standard and a Ca index concentration value of 35% (Brodbeck et al. 2023). USGS MAPS‐4 (synthetic bone) was used as a primary calibration standard and NIST 610 was used as an external secondary reference standard. Standards were analyzed hourly in triplicate for 60s. Because signals for ^137^Ba and ^138^Ba were virtually identical (Pearson *R* = 0.97: *t*‐test; *t* = 0.57, *P* (*T* ≤ *t*) two‐tail > 0.05), we use the higher abundance isotope for plotting and statistical analysis.

## 3 Results

The photographs provided by the fisher (Figure 2a–c) allowed us to confirm that it was a Bull Shark, although we were not able to establish the sex of the specimen. On September 14th, 2025, the fisher reported netting another smaller shark recently that he released alive. The fisher also indicated that these two individuals were the first that he had caught while fishing at Lake Gatun, but that he had also recently seen three small dead sharks in the same area where he routinely deploys his nets (between Gamboa and BCI; Figure 1).

**FIGURE 3:**
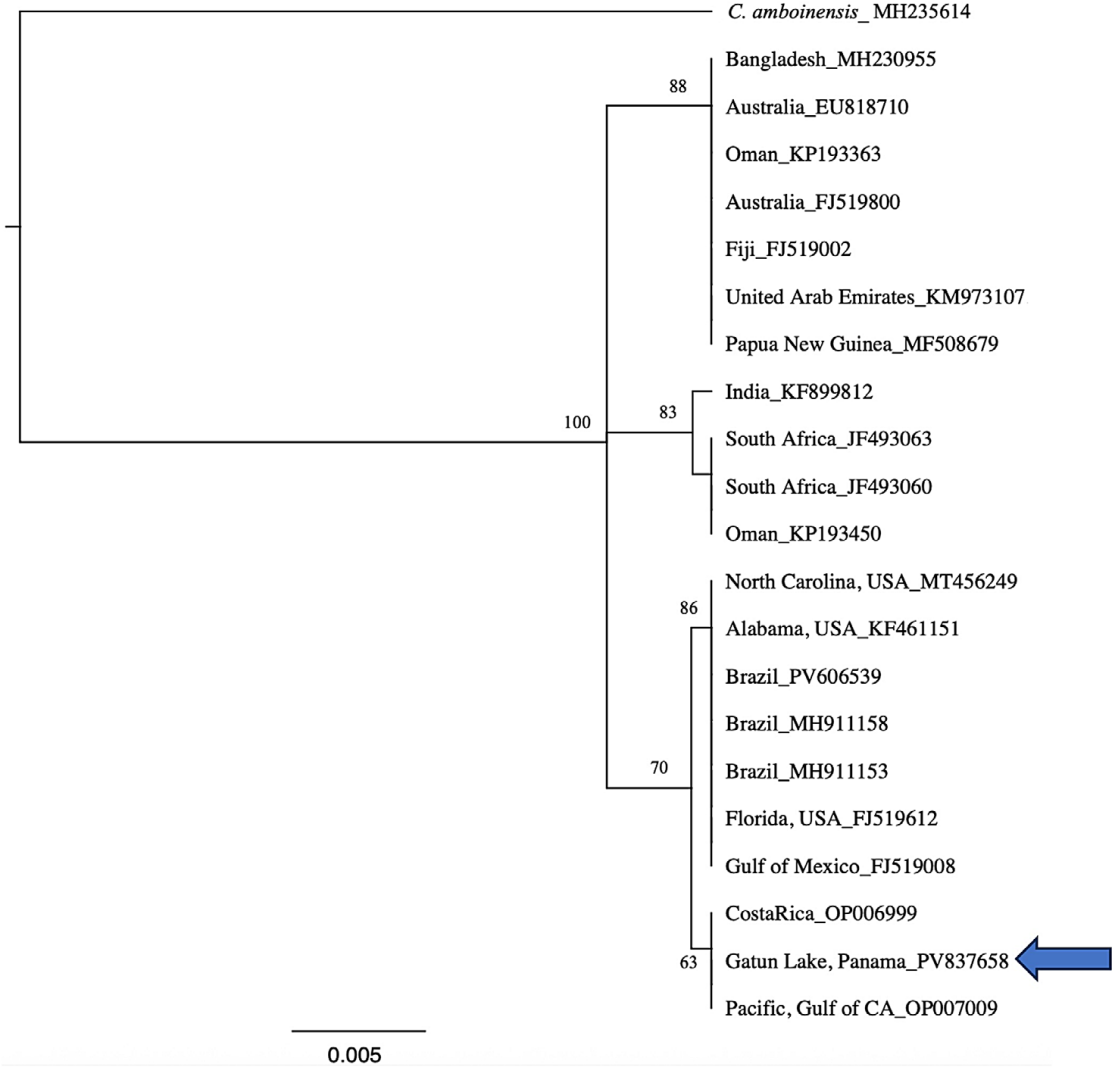
UPGMA tree showing relationship of the Lake Gatun Bull Shark (indicated with an arrow) to other *C. leucas* individuals from throughout the range of the species. The country of origin and GenBank accession numbers are shown for each specimen.

The 655 bp COI sequence that we obtained is most similar to Bull Shark accessions collected on the Pacific side of Costa Rica and the Gulf of California (Figure 3), indicating that this shark originated from the Pacific side of the Panama Canal and had traveled more than 30 km from the Pacific locks to the capture site in the middle of Lake Gatun.

**FIGURE 4:**
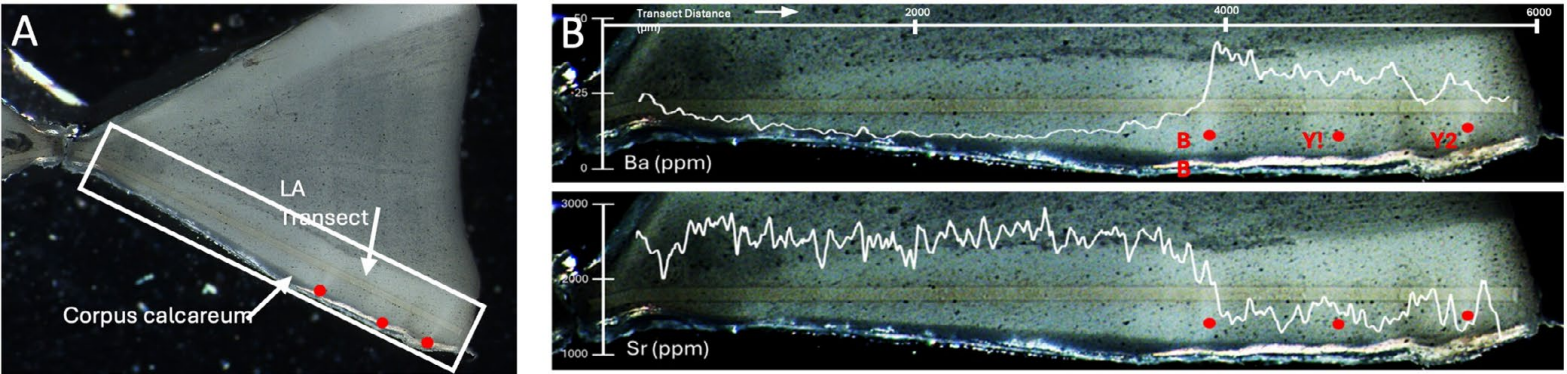
(A) Vertebral section as viewed under transmitted light with the laser ablation transect (LA transect) visible on the corpus calcareum (outlined with rectangle). (B) Lifetime Sr. and Ba values superimposed onto the LA transect. In all images, red dots indicate the birth band (BB) and year‐1 (Y1) and year‐2 (Y2) band pairs.

Using the head to total length relationships in photographs, we estimated the TL of the shark to be ~150 cm. The prepared vertebra had a centrum diameter of 11.95 mm, resulting in an estimated size ~120 cm TL when applied to population demographic data for Bull Sharks in the southern Gulf of Mexico (Cruz‐Martinez et al. 2005), northwest Atlantic Ocean (Natanson et al. 2014), and Reunion Island (Hoarau et al. 2021). Under transmitted light, the birth band and two additional growth bands were clearly visible in the corpus calcareum, indicating the shark was over 2 years old when captured (Figure 4A), consistent with documented age‐length relationships for Bull Sharks (Cruz‐Martinez et al. 2005; Natanson et al. 2014; Hoarau et al. 2021). Based on the available length at maturity estimates for Bull Sharks (males: 157–226 cm TL and females: 180–230 cm TL; Rigby et al. 2021), this specimen was immature.

After the birth band, Sr. and Ba values indicate constant residency in a low salinity environment, less than 2000 ppm and greater than 15 ppm, respectively (Figure 4). Since gestation values are indicative of adult marine residency and the chemistry shift occurs at the birth band, it likely indicates a pupping movement from high to low salinity waters common in *C. leucas* (McMillan et al. 2017; Livernois et al. 2021). However, while it is possible that this individual was pupped in Lake Gatun, juvenile Bull Sharks are capable of long‐distance coastal movements (Edwards et al. 2022). This individual could have been pupped in a tidal river near the locks or further afield and moved into Lake Gatun while minimizing long‐term exposure to high salinity waters.

## 4 Discussion

Prior to this report, the only credible record of any species of shark in the locks or freshwater part of the Panama Canal was that of Hildebrand (1939), who reported the collection of one small specimen of the genus *Carcharhinus* (not identified to species) from the lower (seaward) chamber of Miraflores Locks (Pacific side) in 1937. This collector also noted the presence of three other similar sharks in the same lock, but did not collect any of them. The skin of that specimen is stored at the Smithsonian National Museum of Natural History (USNM) and identified in its catalog (Accession no. 13961; USNM 127134) as a *C. leucas* . Based on our review of original collection documents at USNM, it is evident that Bigelow and Schroeder (1948) examined that specimen and reported it as a ca. 1137 mm female of *C. leucas* . That specimen is the only shark collected from a lock or freshwater part of the Panama Canal recorded in the USNM online Catalog and neither of the two major global aggregators of such data, GBIF (https://www.gbif.org/) nor OBIS (https://obis.org/), have any other records of sharks of any species from those parts of the Canal. Although a compilation of shark attacks in Panama (Averza Colamarco 2003) cited a 1935 New York Times report of a lethal attack on a boy by an unidentified shark in the Culebra Cut, in the freshwater section of the Panama Canal, the article refers to Culebra as being on the Atlantic coast of Panama (Figure S1). Since that attack was associated with dynamite fishing, an activity that would never have been permitted inside the Culebra Cut, which is a heavily restricted part of the Canal, it most likely occurred near the mouth of the Culebra River, ~70 km east of the Atlantic entrance to the Canal.

The documentation of multiple individuals in the seaward lock of the Canal in 1937 demonstrates that immature Pacific *C. leucas* have been at the outer edge of the freshwater part of the Canal since long before the specimen that we analyze here was caught inside Lake Gatun. Observations by a Panama Canal ship pilot indicate that sharks repeatedly occur around the seaward entrances to the Pacific side locks. A video taken at night by that pilot in July 2025 shows two members of an aggregation of 5–6 small sharks swimming ~150 m outside the seaward entrance of the new Cocolí locks at the Pacific end of the Canal (Video S1), which would facilitate entrance to the Canal and Lake Gatun by this top‐order predator.

Nearly 10 years after the new lock systems of the expanded Panama Canal became operational in 2016, the fish communities inside Lake Gatun have undergone a major reconfiguration (Castellanos‐Galindo et al. 2020). From a formerly freshwater‐dominated fish community, catches in many locations of this lake are now dominated by fish species of marine origin (Schreiber et al. 2023; Castellanos‐Galindo et al. 2025). Given the high tolerance of the Bull Shark to low salinities and its well‐known propensity to travel up rivers (Gausmann 2021), the presence of this species inside the freshwater portion of the Panama Canal is not surprising. The new lock system at Cocolí (Figure 1), which provides direct access to Lake Gatun, could be facilitating the incursion of a variety of euryhaline species, including top predators such as the Bull Shark from the Pacific Ocean, a situation that requires further evaluation. The capture of a Bull Shark from the Eastern Pacific in the middle of the freshwater lake of the Panama Canal also indicates the potential for increased contact between Pacific and Atlantic Bull Shark populations. The possible interoceanic movement across the Panama Canal by this species and others suggests that we are potentially witnessing a massive human‐facilitated interoceanic dispersal process, in which ca. 3.5 million years of separation of eastern Pacific and Atlantic marine fauna is disintegrating. While the ecological and evolutionary consequences of such contact are unknown, the increased possibility of it happening may impact the integrity of Pacific and Atlantic coastal marine communities in the tropical Americas. Given that other human impacts on the ocean can act synergistically making ecosystem recovery harder (Jackson 2008), human modification of the physical environment leading to altered dispersal patterns may create further challenges in the Anthropocene.

## Author Contributions

**Gustavo A. Castellanos‐Galindo:** conceptualization (lead), investigation (equal), project administration (lead), resources (equal), supervision (equal), visualization (equal), writing – original draft (lead). **D. Ross Robertson:** conceptualization (lead), formal analysis (equal), investigation (lead), project administration (lead), supervision (lead), writing – original draft (lead). **Victor Bravo:** conceptualization (equal), data curation (supporting), investigation (supporting), methodology (supporting), resources (supporting), writing – review and editing (equal). **Kristin Saltonstall:** conceptualization (equal), data curation (equal), formal analysis (equal), funding acquisition (equal), investigation (equal), methodology (equal), project administration (equal), resources (equal), software (equal), supervision (equal), validation (equal), visualization (equal), writing – original draft (equal), writing – review and editing (equal). **Phillip Sanchez:** data curation (equal), formal analysis (equal), investigation (equal), methodology (equal), resources (equal), software (equal), validation (equal), visualization (equal), writing – review and editing (equal). **Lucia Morales:** data curation (supporting), formal analysis (supporting), investigation (supporting), methodology (supporting), software (supporting), validation (supporting), visualization (supporting). **Richard Cahill:** data curation (supporting), investigation (supporting), methodology (supporting), visualization (supporting), writing – review and editing (supporting). **Mark E. Torchin:** conceptualization (equal), data curation (equal), funding acquisition (equal), investigation (lead), methodology (equal), project administration (lead), resources (lead), software (equal), supervision (lead), validation (equal), visualization (equal), writing – original draft (equal), writing – review and editing (equal).

## Funding

This work was supported by Deutsche Forschungsgemeinschaft, CA 2261/3‐1, project number 471823073. Smithsonian Institution Barcode Network, FY24 Award Cycle.

## Conflicts of Interest

The authors declare no conflicts of interest.

## Supporting information


**Figure S1:** New York Times news article from July 6, 1935 (in blue square) documenting a shark attack in a locality named Culebra on the Atlantic coast of Panama.

**Data S1:** ece373114‐sup‐0002‐Supinfo.tif.

**Video S1:** ece373114‐sup‐0003‐VideoS1.mp4.

## Data Availability

The DNA sequence obtained for this article is available in the NCBI GenBank nucleotide database and can be accessed with accession number PV837658.
