# Supplementary material for: First Confirmed Record of a Bull Shark in Lake Gatun, the Freshwater Body of the Panama Canal
Source: Ecol Evol. 2026 Feb 23;16(2):e73114. doi: 10.1002/ece3.73114 (PMC12928108; doi:10.1002/ece3.73114)
Supplement: Supplementary file 1 — Figure S1: New York Times news article from July 6, 1935 (in blue square) documenting a shark attack in a locality named Culebra on the Atlantic coast of Panama. [file ECE3-16-e73114-s001.docx]

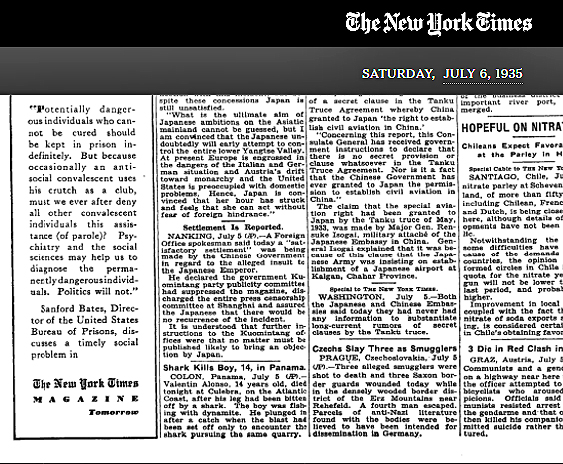


**Figure S1.** New York Times news article from July 6, 1935 (in blue square) documenting a shark attack in a locality names Culebra on the Atlantic coast of Panama. The location of this report was mistakenly assigned to Culebra Cut within the Panama Canal in a later scholarly article.
