# Supplementary figures and images for: First Confirmed Record of a Bull Shark in Lake Gatun, the Freshwater Body of the Panama Canal
Source: Ecol Evol. 2026 Feb 23;16(2):e73114. doi: 10.1002/ece3.73114 (PMC12928108; doi:10.1002/ece3.73114)

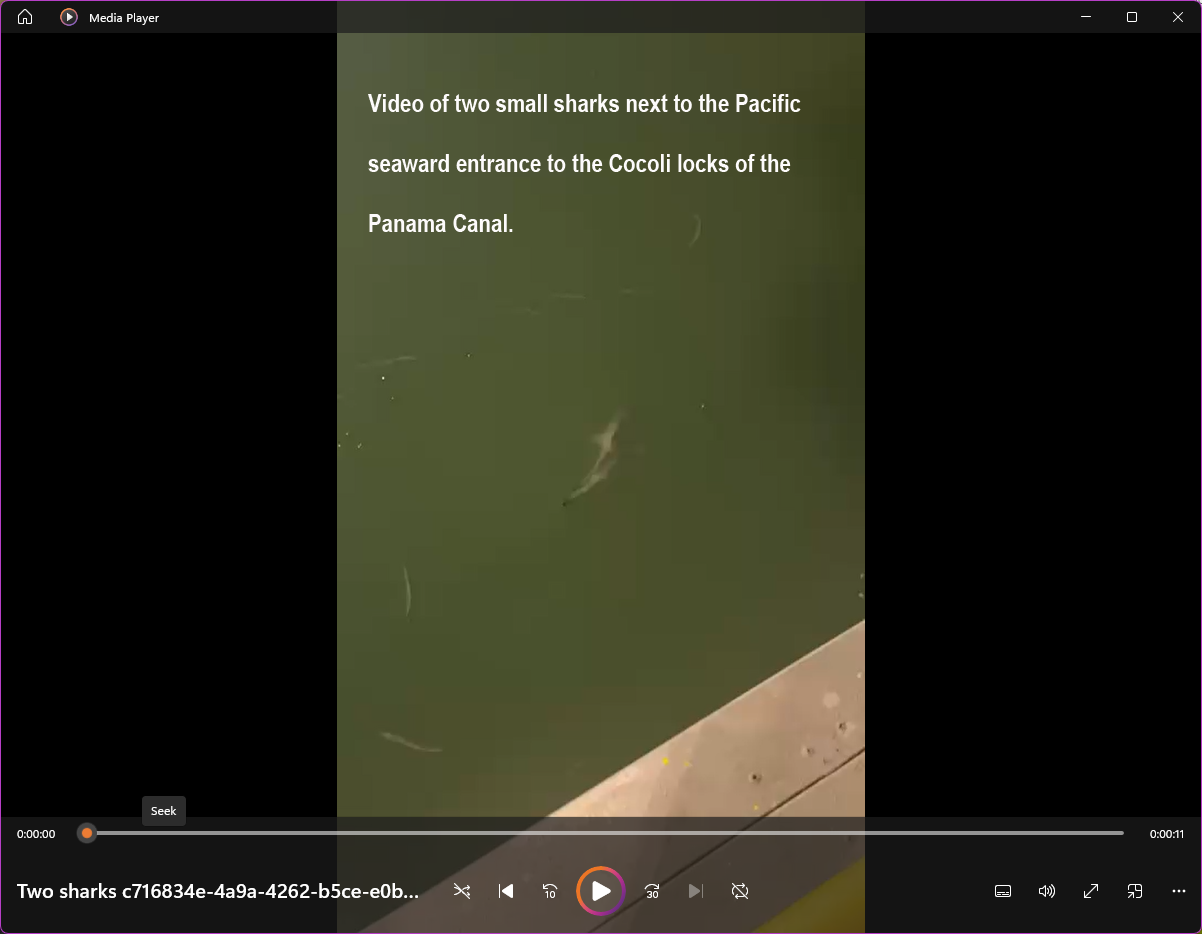

Supplement: Supplementary file 2 — Data S1: ece373114‐sup‐0002‐Supinfo.tif. [file ECE3-16-e73114-s002.tif]
